# Essential medicines for cancer: WHO recommendations and national priorities

**DOI:** 10.2471/BLT.15.163998

**Published:** 2016-07-05

**Authors:** Jane Robertson, Ronald Barr, Lawrence N Shulman, Gilles B Forte, Nicola Magrini

**Affiliations:** a School of Medicine and Public Health, The University of Newcastle, Newcastle, Australia.; b Departments of Pediatrics, Pathology and Medicine, McMaster University, Hamilton, Canada.; c Center for Global Cancer Medicine, University of Pennsylvania, Philadelphia, United States of America.; d Department of Essential Medicines and Health Products, World Health Organization, 20 avenue Appia, 1211 Geneva 27, Switzerland.

## Abstract

**Objective:**

To examine, for essential anti-cancer medicines, the alignment of national lists of essential medicines and national reimbursable medicines lists with the World Health Organization’s (WHO’s) Model Lists.

**Methods:**

National medicine lists for 135 countries with per-capita gross national incomes below 25 000 United States dollars in 2015 were compared with WHO’s 2013 and 2015 Model Lists of Essential Medicines. Correlations between numbers of anti-cancer medicines included in national lists and gross national income (GNI), government health expenditure and number of physicians per 1000 population were evaluated.

**Findings:**

Of the 25 anti-cancer medicines on the 2013 Model List and the 16 added via the 2015 revision of the Model List, 0–25 (median: 17) and 0–15 (median: 3) appeared in national lists, respectively. There was considerable variability in these numbers within and between World Bank income groups. Of the 16 new medicines included in the 2015 Model List, for example, 0–10 (median: 1) and 2–15 (median: 10) were included in the national lists of low-income and high-income countries, respectively. The numbers of these new medicines included in national lists were significantly correlated (*P* ≤ 0.0001) with per-capita GNI (*r* = 0.45), per-capita annual government health expenditure (*r* = 0.33) and number of physicians per 1000 population (*r* = 0.48). Twenty-one countries (16%) included the targeted anti-cancer medicines imatinib, rituximab and trastuzumab in their national lists.

**Conclusion:**

Substantial numbers of anti-cancer medicines are included in national lists of low- and middle-income countries but the availability, affordability, accessibility and administration feasibility of these medicines, at country-level, need assessment.

## Résumé

**Objectif:**

Examiner, en ce qui concerne les médicaments essentiels pour traiter le cancer, la conformité des listes nationales de médicaments essentiels et des listes nationales de médicaments remboursables avec les Listes modèles de l'Organisation mondiale de la Santé (OMS).

**Méthodes:**

Les listes nationales de médicaments de 135 pays dont le revenu national brut par habitant était inférieur à 25 000 dollars des États-Unis en 2015 ont été comparées aux Listes modèles de médicaments essentiels de 2013 et 2015 de l'OMS. Nous avons mis en corrélation le nombre de médicaments pour traiter le cancer inclus dans les listes nationales avec le revenu national brut (RNB), les dépenses publiques de santé et le nombre de médecins pour 1000 habitants.

**Résultats:**

Sur les 25 médicaments pour traiter le cancer inclus dans la Liste modèle de 2013, 0–25 (médiane: 17) apparaissaient sur les listes nationales, et sur les 16 ajoutés lors de la révision de la Liste modèle de 2015, 0–15 (médiane: 3) figuraient sur les listes nationales. Ces chiffres étaient très variables au sein des groupes de revenus de la Banque mondiale et entre eux. Ainsi, sur les 16 nouveaux médicaments inclus dans la Liste modèle de 2015, 0–10 (médiane: 1) apparaissaient sur les listes nationales des pays à faible revenu et 2–15 (médiane: 10) sur celles des pays à revenu élevé. Le nombre de nouveaux médicaments figurant sur les listes nationales était significativement corrélé (P ≤ 0,0001) avec le RNB par habitant (r = 0,45), les dépenses annuelles de santé par habitant engagées par le gouvernement (r = 0,33) et le nombre de médecins pour 1000 habitants (r = 0,48). L'imatinib, le rituximab et le trastuzumab, trois médicaments utilisés pour traiter le cancer, apparaissaient sur la liste nationale de vingt-et-un pays (16%).

**Conclusion:**

Un nombre important de médicaments pour traiter le cancer est inclus dans les listes nationales des pays à revenu faible et intermédiaire, mais il convient d'évaluer la disponibilité, le caractère économiquement abordable, l'accessibilité et la faisabilité administrative de ces médicaments au niveau des pays.

## Resumen

**Objetivo:**

Examinar, en términos de los medicamentos esenciales contra el cáncer, la coherencia de las listas nacionales de medicamentos esenciales y las listas nacionales de fármacos reembolsables con las Listas Modelo de la Organización Mundial de la Salud (OMS).

**Métodos:**

En 2015, se compararon las listas nacionales de medicamentos de 135 países con un producto interior bruto per cápita inferior a 25 000 dólares estadounidenses con las Listas Modelo de Medicamentos Esenciales de la OMS de 2013 y 2015. Se evaluaron las correlaciones entre los números de medicamentos contra el cáncer incluidos en las listas nacionales y el producto interior bruto (PIB), el gasto sanitario público y el número de médicos por cada 1 000 habitantes.

**Resultados:**

De los 25 medicamentos contra el cáncer de la Lista Modelo de 2013 y los 16 que se añadieron en la revisión de 2015 de la Lista Modelo, 0–25 (promedio: 17) y 0–15 (promedio: 3) aparecían en las listas nacionales, respectivamente. Hubo una variabilidad considerable en estos números dentro y entre los grupos de ingresos del Banco Mundial. De los 16 nuevos medicamentos incluidos en la Lista Modelo de 2015, por ejemplo, se incluyeron 0–10 (promedio: 1) y 2–15 (promedio: 10) en las listas nacionales de países con ingresos bajos y con ingresos altos, respectivamente. Los números de estos nuevos medicamentos incluidos en las listas nacionales tenían una gran correlación (P ≤ 0,0001) con el PIB per cápita (r = 0,45), el gasto sanitario público anual per cápita (r = 0,33) y el número de médicos por cada 1 000 habitantes (r = 0,48). Veintiún países (16%) incluían en sus listas nacionales los medicamentos contra el cáncer imatinib, rituximab y trastuzumab.

**Conclusión:**

Se han incluido numerosos medicamentos contra el cáncer en listas nacionales de países con ingresos bajos y medios, aunque la disponibilidad, asequibilidad, acceso y viabilidad de administración de los mismos necesitan una evaluación a nivel del país.

## ملخص

**الغرض:**

بالنسبة للأدوية الأساسية المقاومة للسرطان، لدراسة مدى توازي القوائم الوطنية للأدوية الضرورية وقوائم الأدوية الوطنية مستحقة الدفع مع قوائم الأدوية الهامة والأساسية لمنظمة الصحة العالمية (WHO).

**الطريقة:**

تمت مقارنة قوائم الأدوية الوطنية لـ 135 بلدًا بإجمالي الدخل القومي للفرد الواحد الأقل من 25000 دولارًا أمريكيًا في عام 2015 مع قوائم الأدوية الأساسية والهامة لمنظمة الصحة العالمية في عامي 2013 و2015. وتم تقييم الارتباط بين عدد من الأدوية المقاومة للسرطان المدرجة في القوائم الوطنية وإجمالي الدخل القومي (GNI)، والنفقات الصحية الحكومية وعدد الأطباء لكل 1000 نسمة.

**النتائج:**

من بين 25 دواءً مقاوم للسرطان مذكور في قائمة الأدوية الأساسية والهامة لعام 2013 و16 دواءً مضافًا عبر مراجعة قائمة الأدوية الأساسية والهامة في عام 2015، ظهر 0–25 (المتوسط: 17) و0–15 (المتوسط: 3) في القوائم الوطنية، على التوالي. كان هناك تباينًا كبيرًا في هذه الأرقام داخل وبين فئات الدخل في البنك الدولي. ومن بين 16 دواءً جديدًا تم ضمها في قائمة الأدوية الهامة والأساسية لعام 2015، على سبيل المثال، 0–10 (المتوسط: 1) و2–15 (المتوسط: 10) تم ضمها في القوائم الوطنية للبلدان منخفضة الدخل ومرتفعة الدخل، على التوالي. تم ربط أعداد هذه الأدوية الجديدة المشمولة في القوائم الوطنية بشكلٍ كبير (الاحتمال ≤ 0.0001) مع إجمالي الدخل القومي للفرد الواحد (درجة الارتباط = 0.45)، والنفقات الصحية الحكومية السنوية للفرد الواحد (درجة الارتباط = 0.33)، وعدد الأطباء لكل 1000 نسمة (درجة الارتباط = 0.48). وقامت إحدى وعشرين بلدًا (%16) بضم الأدوية المقاومة للسرطان المستهدفة وهي imatinib وrituximab وtrastuzumab في قوائمها الوطنية.

**الاستنتاج:**

تم ضم أعداد كبيرة من الأدوية المقاومة للسرطان في القوائم الوطنية في البلدان منخفضة ومتوسطة الدخل، ولكن عوامل توافر هذه الأدوية والقدرة على دفع ثمنها وإمكانية الوصول إليها وقابلية تناولها، تحتاج إلى أن يتم تقييمها على المستوى القطري.

## 摘要

**目的:**

审查国家基本药物目录和国家报销药品目录中的基本抗癌药物与世界卫生组织 (WHO) 基本药物标准清单中的基本抗癌药物的符合度。

**方法:**

将 2015 年人均国民总收入低于 25 000 美元的 135 个国家的国家药物目录与世界卫生组织 2013 年和 2015 年的基本药物标准清单进行比较。 我们分别评估了国家目录中包含的抗癌药物的数目与国民总收入 (GNI)、政府卫生支出以及每 1000 人所配备医生数目之间的相关性。

**结果:**

在 2013 年标准清单中的 25 种抗癌药物以及 2015 年标准清单修订版中新添的 16 种药物中,分别有 0–25 种(中值: 17)和 0–15 种(中值: 3)出现在国家目录中。 这些数据在不同的世界银行收入组内部和相互之间呈现出很高的可变性。 例如, 2015 年标准清单中包含的 16 种新药物中, 0–10 种(中值: 1)和 2–15 种(中值: 10)分别出现在低收入和高收入国家的国家目录中。 国家目录中包含的这些新药的数目与人均国民总收入 (GNI) (r = 0.45)、人均政府年度卫生支出 (r = 0.33) 以及每 1000 人所配备的医生数目 (r = 0.48) 呈显著相关关系 (P ≤ 0.0001)。 21 个国家 (16%) 的国家目录中含有目标抗癌药物伊马替尼、利妥昔单抗、曲妥单抗。

**结论:**

中低收入国家的国家目录中包含大量抗癌药物,但是还需要在国家层面对这些药物的可获得性、经济承受性、可及性以及管理可行性加以评估。

## Резюме

**Цель:**

Изучить степень согласованности национальных перечней основных лекарств и национальных перечней возмещаемых лекарств с примерными перечнями Всемирной организации здравоохранения (ВОЗ) относительно того, что касается основных противораковых лекарственных препаратов.

**Методы:**

Национальные перечни лекарственных средств для 135 стран, в которых валовой национальный доход на душу населения составил менее 25 000 долларов США в 2015 году, сравнили с примерными перечнями основных лекарств ВОЗ 2013 и 2015 годов. Были определены корреляции между количеством противораковых лекарств, включенных в национальные перечни, и валовым национальным доходом (ВНД), государственными расходами в области здравоохранения и количеством врачей на 1000 жителей.

**Результаты:**

Из 25 противораковых препаратов, указанных в примерном перечне 2013 года, и 16 добавленных в редакцию примерного перечня, выпущенную в 2015 году, от 0 до 25 (среднее значение — 17) и от 0 до 15 (среднее значение — 3) соответственно присутствовали в национальных перечнях. Были выявлены значительные различия в этих показателях как между группами стран по уровню доходов по классификации Всемирного банка, так и внутри них. Например, из 16 новых лекарственных средств, включенных в примерный список 2015 года, от 0 до 10 (среднее значение — 1) и от 2 до 15 (среднее значение — 10) были включены в национальные перечни стран с низким и высоким уровнем доходов соответственно. Количества этих новых лекарств, включенных в национальные перечни, демонстрировали значительную корреляцию (P ≤ 0,0001) с ВНД на душу населения (r = 0,45), ежегодными государственными расходами в области здравоохранения на душу населения (r = 0,33) и количеством врачей на 1000 жителей (r = 0,48). Двадцать одна страна (16%) включила таргетные противораковые препараты — иматиниб, ритуксимаб и трастузумаб — в свои национальные перечни.

**Вывод:**

Значительное количество противораковых лекарственных средств включено в национальные перечни стран с низким и средним уровнем дохода, однако требуется оценка наличия, доступности (в том числе ценовой) и применимости этих лекарств на уровне отдельных стран.

## Introduction

Effective treatment of cancer relies on early detection, accurate diagnosis and access to surgery, chemotherapy and/or radiotherapy. The provision of affordable access to cytotoxic medicines is a major challenge in the care of patients with cancer, especially in resource-poor settings.^1^^,^^2^ By developing its Model Lists of Essential Medicines, the World Health Organization (WHO) aims to help countries prioritize and select the medicines to include in their national essential medicines lists and, increasingly, national reimbursable medicines lists.^3^ WHO published its first Model List in 1977. Although the general list has since been updated every two years – based on the recommendations of an expert committee – the consideration of essential medicines for cancer has been less regular, with the most substantial reviews occurring in 1984, 1994 and 1999.^4^^–^^6^

Given the increasing burden of cancer globally and the very high cure rates now being achieved for some cancers, there is a strong argument for considering anti-cancer drugs as essential medicines.^7^ In 2011, the relevant expert committee specifically reviewed medicines for acute lymphoblastic leukaemia, Burkitt lymphoma and Wilms tumour in children – adding several medicines to WHO’s 2011 Model List of Essential Medicines for Children and identifying the diseases for which the medicines were listed.^8^ In 2015, the expert committee undertook a comprehensive review of essential medicines for cancer, in both adults and children, as part of an international consultative process coordinated by the Union for International Cancer Control.^9^ The committee focused on common or rare cancers for which there was evidence of relevant clinical benefit, particularly the potential for cure or long-term remission. It considered the relative contribution of single-agent or multi-drug regimens, surgery and radiation to treatment outcomes and took account of the feasibility of the use of each therapeutic option – particularly in low- and middle-income countries.^10^ The committee’s discussions resulted in a comprehensive review of treatments for 29 cancer indications and a request to add 21 cytotoxic medicines and one supportive therapy – i.e. granulocyte colony stimulating factor – to WHO’s lists of essential medicines for cancer (Table 1). To provide treatment options for Ewing sarcoma, germ cell tumours, Hodgkin lymphoma, osteosarcoma, retinoblastoma and rhabdomyosarcoma, the committee recommended that carboplatin, cisplatin, dacarbazine, etoposide and ifosfamide be added to the 2015 Model List of Essential Medicines for Children. It also recommended that 16 new anti-cancer medicines be added to the 2015 Model List of Essential Medicines – for the treatment of adults. The expert committee declined to include arsenic trioxide, dasatinib, diethylstilboestrol, erlotinib, gefitinib and nilotinib on the 2015 Model List of Essential Medicines, though each of these agents had previously been identified as priority medicines by the relevant working group. The reasons for these rejections are articulated in the 2015 report of the expert committee.^11^

**Table 1:** Essential medicines for cancer on the national essential medicines lists or national reimbursable medicines lists of 135 countries, 2015

Medicine	No. of countries listing medicine (%)
**On WHO 2013 Model List**	
Asparaginase	68 (50)
Bleomycin	94 (70)
Calcium folinate	84 (62)
Carboplatin	73 (54)
Chlorambucil	84 (62)
Cyclophosphamide	120 (89)
Cytarabine	88 (65)
Dacarbazine	73 (54)
Dactinomycin	67 (50)
Daunorubicin	55 (41)
Docetaxel	56 (42)
Doxorubicin	99 (73)
Etoposide	84 (62)
Fluorouracil	109 (81)
Hydroxycarbamide	88 (65)
Ifosfamide	60 (44)
Mercaptopurine	87 (64)
Mesna	53 (39)
Methotrexate	128 (95)
Paclitaxel	65 (48)
Procarbazine	62 (46)
Tamoxifen	112 (83)
Tioguanine	36 (27)
Vinblastine	83 (61)
Vincristine	111 (82)
**Added to WHO Model List via 2015 revision**	
All-trans retinoic acid^a^	19 (14)
Aromatase inhibitors^b^	50 (37)
Bendamustine	1 (0.7)
Bicalutamide	38 (28)
Capecitabine	49 (36)
Cisplatin	97 (72)
Fludarabine	40 (30)
Gemcitabine	46 (34)
Granulocyte colony stimulating factors^c^	63 (47)
Imatinib	40 (30)
Irinotecan	41 (30)
Leuprolin class^d^	53 (39)
Oxaliplatin	49 (36)
Rituximab	34 (25)
Trastuzumab	26 (19)
Vinorelbine	37 (27)
**Other medicines**^e^	
Arsenic trioxide	2 (1)
Dasatinib	10 (7)
Diethylstilboestrol	6 (4)
Erlotinib	14 (10)
Gefitinib	9 (7)
Nilotinib	12 (9)

WHO: World Health Organization.

^a^ In capsule or tablet form only. Also known as tretinoin.

^b^ Anastrazole, exemestane and/or letrozole.

^c^ Filgrastim, lenograstim and/or pegfilgrastim.

^d^ Goserelin, leuprolin and/or triptorelin.

^e^ Proposed for inclusion in the 2015 WHO Model List but excluded by the relevant expert committee.

The level of alignment, in terms of anti-cancer medicines, between national essential medicines lists and WHO’s Model Lists has rarely been investigated. A review of 76 national essential medicines lists revealed considerable variation in the listing of anti-cancer medicines by geographical region, socioeconomic status and burden of disease, with few of the lists including newer targeted therapies such as monoclonal antibodies and tyrosine kinase inhibitors.^12^ A study of treatments for breast cancer revealed that fewer than 10% of the 75 countries investigated included trastuzumab in their national essential medicines lists.^13^

We recently evaluated the level of alignment, in terms of anti-cancer medicines, between national essential medicines lists or national reimbursable medicines lists – hereafter grouped together under the term national medicines lists – and WHO’s 2013 and 2015 Model Lists of Essential Medicines for adults and children. One of our aims was to determine the degree of uptake of – and perceived need for – anti-cancer medicines. We evaluated the correlations between the numbers of anti-cancer medicines included in the national medicines lists and several financial and workforce characteristics.

## Methods

National medicines lists were obtained from several sources, including two WHO websites,^14^^,^^15^ WHO country offices and – where updated lists were known to the authors but did not appear on WHO websites – Internet searches. If, for a particular country, separate lists existed for adults and children, we extracted and combined the names of the anti-cancer medicines on the two lists. Data were extracted independently by two authors. Any discrepancies were resolved by consensus and checking the relevant source documents. Translations were obtained for the lists that used the Cyrillic alphabet – i.e. those of Belarus, the Russian Federation, Tajikistan, Ukraine and Uzbekistan – and the list for Mongolia. No distinction was made on the basis of the form and strength of any medicine.

Information on per-capita gross national income (GNI) was based on the Atlas method and obtained from the World Bank.^16^ By confining our analysis to lists from countries that had a per-capita GNI for 2015 below 25 000 United States dollars (US$), we aimed to minimize any ceiling effect caused by the wealthier countries having all of the anti-cancer drugs on WHO’s Model Lists included in their national medicines lists. Following the World Bank, we defined countries that had, in 2015, a per-capita GNI of less than US$ 1046 as low-income, US$ 1046–4125 as lower-middle-income, US$ 4126–12 745 as upper-middle-income and more than US$ 12 745 as high-income. For each study country, we obtained data on annual per-capita government health expenditure and number of physicians per 1000 population from WHO’s Global Observatory.^11^

We report the proportions of countries that included, on their national medicines list or lists, each of the essential anti-cancer medicines on the 2013 and 2015 Model Lists and we report the median numbers of such medicines listed overall, by time since the national list was last updated, by World Bank income group and by WHO region. Correlations between the median numbers of anti-cancer medicines listed and per-capita GNI, physician density and per-capita government expenditure on health were evaluated, as Pearson’s correlation coefficients (*r*), using Excel (Microsoft, Redmond, United States of America) spreadsheets.

## Results

Information was available from 135 countries that had per-capita GNI for 2015 below US$ 25 000 (Box 1). Of the 135 study countries, 26 were low-income, 42 were lower-middle-income, 44 were upper-middle-income and 20 were high-income. Three countries could not be classified into an income group. At the time of our review, in June 2015, the median year of release of the most recently available national medicines list was 2010 (range: 2004–2014). Forty-one (30%) of the countries had updated their national medicines lists since 2012.

Box 1 **Countries^a^ included in the study and year that the latest national essential medicines list or national reimbursable medicines list was issued, 2015**

**WHO African Region**

Algeria (2007), Benin (2013), Botswana (2012), Burkina Faso (2012), Burundi (2012), Cabo Verde (2014), Cameroon (2010), Central African Republic (2009), Chad (2007), Congo (2013), Côte d’Ivoire (NA), Democratic Republic of the Congo (2010), Eritrea (2010), Ethiopia (2010), Gabon (NA), Ghana (2010), Guinea (2012), Kenya (2010), Lesotho (2005), Liberia (2011), Madagascar (2008), Malawi (2009), Mali (2012), Mauritania (2008), Mozambique (2010), Namibia (2008), Nigeria (2010), Rwanda (2010), Senegal (2013), Seychelles (2010), South Africa (2012), Swaziland (2011), Togo (2012), Uganda (2012), United Republic of Tanzania (2013), Zambia (2013) and Zimbabwe (2011).

**WHO Region of the Americas**

Argentina (2005), Barbados (2011), Belize (2009), Bolivia (Plurinational State of) (2011), Brazil (2010), Chile (2005), Colombia (2011), Costa Rica (2010), Cuba (2012), Dominica (2006), Dominican Republic (2005), Ecuador (2013), El Salvador (2009), Grenada (2006), Guyana (2009), Haiti (2012), Honduras (2009), Jamaica (2008), Mexico (2011), Nicaragua (2011), Paraguay (2009), Peru (2012), Saint Kitts and Nevis (2006), Saint Lucia (2006), Saint Vincent and the Grenadines (2010), Suriname (2014), Trinidad and Tobago (2010), Uruguay (2011) and Venezuela (Bolivarian Republic of) (2004).

**WHO South-East Asia Region**

Bangladesh (2008), Bhutan (2012), Democratic People's Republic of Korea (2012), India (2011), Indonesia (2011), Maldives (2011), Myanmar (2010), Nepal (2011), Sri Lanka (2009), Thailand (2012) and Timor-Leste (2010).

**WHO European Region**

Albania (2011), Armenia (2010), Belarus (2012), Bosnia and Herzegovina (2009), Bulgaria (2009), Croatia (2010), Czechia (2012), Estonia (2012), Georgia (2007), Kyrgyzstan (2009), Latvia (2012), Lithuania (2012), Malta (2008), Montenegro (2011), Poland (2012), Portugal (2011), Republic of Moldova (2011), Romania (2012), Russian Federation (2014), Serbia (2010), Slovakia (2012), Slovenia (2010), Tajikistan (2009), The former Yugoslav Republic of Macedonia (2010), Ukraine (2009) and Uzbekistan (2009).

**WHO Eastern Mediterranean Region**

Afghanistan (2014), Bahrain (2009), Egypt (2012), Iran (Islamic Republic of) (2014), Iraq (2010), Jordan (2014), Lebanon (2014), Morocco (2008), Pakistan (2013), Somalia (2006), Sudan (2007), Syrian Arab Republic (2008), Tunisia (2008) and Yemen (2009).

**WHO Western Pacific Region**

Cambodia (2012), China (2012), Cook Islands (2007), Fiji (2013), Kiribati (2009), Malaysia (2010), Marshall Islands (2007), Mongolia (2009), Nauru (2010), Niue (2006), Palau (2006), Papua New Guinea (2012), Philippines (2008), Solomon Islands (2010), Tonga (2007), Tuvalu (2010), Vanuatu (2007) and Viet Nam (2008).

NA: not available; WHO: World Health Organization.^a^ For 20 otherwise eligible countries – Antigua and Barbuda, Azerbaijan, Bahamas, Comoros, Equatorial Guinea, Gambia, Greece, Guatemala, Guinea-Bissau, Hungary, Kazakhstan, Lao People's Democratic Republic, Libya, Mauritius, Micronesia (Federated States of), Panama, Samoa, Sao Tome and Principe, Turkey and Turkmenistan – neither a national essential medicines list nor a national reimbursable medicines list could be identified. Another three countries – Angola, Djibouti and Sierra Leone – were excluded because their national essential medicines lists only covered primary health care or health kits.

The percentage of countries listing each of the 25 anti-cancer medicines on the 2013 Model List, each of the 16 anti-cancer medicines added to the Model List via the 2015 revision and each of the anti-cancer medicines rejected by the expert committee in 2015 varied widely (Table 1). A median of 3 (range: 0–15) of the 16 anti-cancer medicines added to the Model List via the 2015 revision was included in the national medicines lists we investigated. However, the national medicines lists of each of 33 study countries (24%) included at least 10 of these 16 medicines (Table 2). The numbers of anti-cancer drugs that appeared on national medicines lists differed considerably across the World Bank income groups (Table 2). For example, of the 16 anti-cancer medicines added to the Model List via the 2015 revision, only a median of 1 (range: 0–10) was included in the national lists of the low-income countries we studied whereas the corresponding value for the high-income study countries was 10 (range: 2–15).

**Table 2:** Median numbers of essential medicines for cancer, from the World Health Organization’s Model Lists, on the national essential medicines lists or national reimbursable medicines lists of 135 countries, 2015

Country	Median no. listed (range)	
	Of 25 anti-cancer medicines on 2013 WHO Model List	Of 16 anti-cancer medicines added to WHO Model List via 2015 revision
All (*n* = 135)	17 (0–25)	3 (0–15)
With national medicines list updated since 2012 (*n* = 41)	18 (0–25)	5 (0–15)
**Income group**		
Low-income (*n* = 28)	9 (0–23)	1 (0–10)
Lower-middle-income (*n* = 44)	18 (1–25)	2 (0–14)
Upper-middle-income (*n* = 42)	19 (0–25)	7 (0–15)
High-income (*n* = 18)	20 (8–25)	10 (2–15)
**WHO Region**		
Africa (*n* = 37)	13 (1–23)	1 (0–14)
Americas (*n* = 29)	19 (3–25)	6 (0–15)
South-East Asia (*n* = 11)	21 (2–24)	1 (0–13)
Europe (*n* = 26)	18.5 (1–25)	10 (0–15)
Eastern Mediterranean (*n* = 14)	23.5 (0–25)	6.5 (0–15)
Western Pacific (*n* = 18)	7 (0–25)	0.5 (0–15)
Western Pacific (*n* = 9)^a^	19 (9–25)	2 (1–15)

WHO: World Health Organization.

^a^ Excluding nine Pacific Island countries: Cook Islands, Kiribati, Marshall Islands, Nauru, Niue, Palau, Tonga, Tuvalu and Vanuatu.

There also seemed to be considerable variation between WHO regions. For example, the median number of anti-cancer medicines – on the Model Lists – that appeared on the national medicines lists of the 37 study countries in the African Region was relatively low. However, in terms of the numbers of anti-cancer medicines listed, we found more variability within regions than between them (Table 2). The very low median number of medicines listed in the Western Pacific Region is mostly the result of nine small Pacific Island countries that each included just two to five cytotoxic agents – often just methotrexate and tamoxifen – on their national medicines lists. When we excluded these countries from our calculations, the median number of listed anti-cancer medicines for the Western Pacific Region increased to a value much closer to that recorded for the other regions (Table 2).

Correlations between numbers of anti-cancer medicines listed and per-capita GNI (Table 3) were positive, statistically significant and stronger for the 16 anti-cancer medicines added to the Model List via the 2015 revision (*r* = 0.45; *P* < 0.0001) than for the 25 anti-cancer medicines on the 2013 Model List (*r* = 0.22; *P* = 0.0138). Similar results were seen for the correlations between numbers of anti-cancer medicines listed and physician density – with corresponding *r*-values of 0.48 (*P* < 0.0001) and 0.30 (*P* = 0.0004), respectively. Correlations between numbers of anti-cancer medicines listed and per-capita government health expenditure were also statistically significant for the 16 anti-cancer medicines added to the Model List via the 2015 revision (*r* = 0.33; *P* = 0.0001) – but not for those on the 2013 Model List.

**Table 3:** Correlation of numbers of anti-cancer medicines on national essential medicines lists or national reimbursable medicines lists with corresponding gross national income, annual per-capita government health expenditure and physician density, 135 countries, 2015

Variable	Listing of 25 anti-cancer medicines on 2013 WHO Model List			Listing of 16 anti-cancer medicines added to WHO Model List via 2015 revision	
	*r*	*P*		*r*	*P*
Per-capita gross national income in 2015	0.22	0.0138		0.45	< 0.0001
Per-capita government health expenditure^a^	0.05	0.599		0.33	0.0001
Number of physicians per 1000 population^a^	0.30	0.0004		0.48	< 0.0001

WHO: World Health Organization.

^a^ In the most recent year for which data were available at the time of our study – most frequently 2013.

## Discussion

Many of the 25 cytotoxic agents on the 2013 Model Lists for adults and children appeared on the national medicines lists that we investigated; the lists of each of 50 study countries (37%) had at least 20 such medicines and the lists of each of 13 countries (10%) included all 25. 

At the time of our review, many countries had already updated their national lists to include at least some of the 16 anti-cancer medicines that were added to the 2015 Model List as a result of the 2015 revision. This confirms that the revision of the WHO list was long overdue after more than 20 years of limited review of anti-cancer medicines for adults and modest changes to the list of medicines for children in 2011. None of the six agents identified as priority medicines by a working group but rejected by the expert committee in 2015 was widely included in the national lists.

The relevant expert committee did not recommend inclusion of trastuzumab on the 2013 Model List for adults but agreed that the clinical data supported inclusion of the medicine, as an essential medicine, in health systems that had the capacity to manage breast cancer adequately – including early diagnosis, histopathology, surgery and radiotherapy.^17^ Almost 20% (26) of our study countries had included trastuzumab on their national essential medicines lists before the medicine was included on the 2015 Model List. Fifty (37%) had included aromatase inhibitors – i.e. anastrozole, exemestane and/or letrozole – to their lists. and the national medicines lists of each of 21 countries (16%) listed at least one aromatase inhibitor and trastuzumab. We found higher percentages of countries with medicines listed for breast cancer than previously reported,^13^ due, in part, to the between-study differences in selection criteria, the larger numbers of countries included in our study and our use of more recent versions of the national medicines lists for more than 25 countries.

The inclusion of some expensive anti-cancer and other anti-neoplastic medicines on many national medicines lists raises questions about treatment affordability, particularly in resource-constrained environments. Despite some criticism that WHO paid scant attention to local costs when considering trastuzumab as an essential medicine,^18^ the expert committee did recognize the high cost of trastuzumab in 2013 and suggested challenges in establishing this medicine’s cost–effectiveness. The budget impact of providing trastuzumab may make it unaffordable at the country level. After WHO’s Choosing Interventions that are Cost–Effective method was used to assess a breast cancer programme in Peru, it was suggested that the programme could be improved by changing screening strategies and it was not recommended that trastuzumab be made available to all eligible patients.^19^^,^^20^ However, trastuzumab is known to improve the cure rate for patients with early-stage breast cancer that tests positive for human epidermal growth factor receptor 2 and to prolong survival when used, in combination with chemotherapy, in patients with metastatic breast cancer that tests positive for the same receptor – and, at the time of writing, there is no less costly alternative. This situation highlights the tensions caused by differences, between high-income and low-income countries, in the affordability of high-cost patented cancer medicines and access to potentially life-saving treatments for women. In including trastuzumab on its 2015 Model List of Essential Medicines, WHO has made a strong statement about the medicine’s utility and – as happened with therapy for human immunodeficiency virus – this may promote efforts to reduce the medicine’s costs and so increase its affordability and availability.

The expert committee’s 2015 review of cancer medicines for the WHO Model List was notable in terms of: (i) its method – it was disease- and regimen-based; (ii) its scope – it addressed 29 different cancer types; and (iii) its effect – with 16 anti-cancer agents newly included as essential medicines. Standard and alternative treatment regimens for particular cancers were proposed, with the recognition that costs would be prohibitive in some settings. The four medicines in the so-called CHOP regimen used to treat diffuse large B-cell lymphoma are relatively old and off-patent. Although rituximab is patent-protected and costlier and more difficult to administer than the cyclophosphamide, doxorubicin, prednisone and vincristine used in the standard CHOP regimen, its addition to the regimen increases survival rates.^21^ Rituximab was therefore included on the 2015 Model List. However, where rituximab is not available, the CHOP regimen should still be used since many patients will benefit from its use.

Although relatively expensive, the targeted anti-cancer medicines imatinib, rituximab and trastuzumab have each demonstrated large benefits.^11^ All three of these medicines appeared on the national medicines list or lists of each of 21 (16%) of our study countries – indicating that, at the time of our review, at least 21 countries had already identified these medicines as essential despite their economic impact.

Almost half of our study countries had included one or more of the granulocyte colony-stimulating factors in their national medicines lists. These factors are expensive and, while their use in patients at high risk of developing febrile neutropenia is justified, they can be easily overused. The improved use of such factors was one of five measures identified, by the American Society of Clinical Oncology, as part of the Choosing Wisely initiative for reducing costs without compromising cancer care.^22^

Clinicians, researchers and, more recently, professional societies are engaging in the public debate around the high costs of cancer care overall and of cancer medicines in particular – and the impact of these costs on patients and health-care systems.^23^^–^^25^ The American Society of Clinical Oncology and the European Society of Medical Oncologists have published tools and a value framework to facilitate comparisons – of the relative clinical benefits, side-effects and costs of cancer treatments – that can support discussion between all of the relevant stakeholders, including governments and third-party payers, and shared decision-making between doctors and patients.^26^^,^^27^ In 2015, the expert committee declined to identify an explicit threshold for the magnitude of clinical benefit that might define an essential medicine for cancer – not only because of the inherent complexity but also because of the need for a wider discussion involving all stakeholders.

The delivery of effective cancer services is complex and requires substantial investment in health facilities and technologies and trained health workers who are able to provide care of good quality. We therefore anticipated the observed correlations between the numbers of anti-cancer medicines listed and per-capita GNI, per-capita government health expenditure and physician density.

Our review had several limitations. For example, we made no attempt to investigate national documents or lists applicable to specialist cancer facilities and we confined our review to anti-cancer drugs that appeared on the 2013 Model List and/or the 2015 Model List. We also made no attempt to investigate, for each study country, the actual availability or use of listed medicines. In some settings, medicines on a national list of essential medicines may not be purchased. Even if purchased, their use may be limited or prevented by a weak infrastructure or because the medicines are unaffordable or inaccessible to those who could benefit. The high costs of cancer care contribute to treatment abandonment in low-income and lower-middle-income countries.^28^ If use is influenced by ability to pay, there are important questions about equity of access that must be addressed.

It should be possible to reduce the total costs of cancer care – with little impact on patient outcomes – by reducing medicine prices, increasing the use of imaging and improving end-of-life care.^29^ The preferential funding of cancer medicines over other treatments is controversial and problematic. In the United Kingdom of Great Britain and Northern Ireland, for example, experience with a dedicated fund for cancer therapies that had not been deemed cost–effective has led some to suggest that “the fund has done more harm than good for NHS [National Health System] patients overall. The real winners are the manufacturers, who have been able to sell their drugs to the NHS at unrealistic prices”.^30^ Others have noted that “cancer drug prices are not related to the value of the drug. Prices are based on what has come before and what the seller believes the market will bear”.^31^ Physicians, drug manufacturers, insurers, governments and patients all have a role in bringing about changes to current prices and improving affordability and access.^24^ WHO may have a convening role in bringing together the various stakeholders, promoting the relevant discussions and helping to define thresholds for relevant clinical benefit.

A regularly updated WHO Model List of essential medicines for cancer could provide guidance to countries – particularly low-income and middle-income countries – on the most effective medicines that should be prioritized for procurement and use. New therapies with substantial benefit should be accessible and the Model Lists should support their selective use in appropriate patients. Further Model List revisions should follow the 2015 approach, in which the identification of priorities was based on the estimation of magnitude of benefits and relative toxicities for each disease as well as considerations for diagnosis, treatment, monitoring and costs. Although the engagement of experts on adult and paediatric cancers and organizations such as the Union for International Cancer Control is of importance, stronger health system engagement and new global responses are also required. International collaboration will be required to manage the prices of cancer medicines better, so that all effective and essential treatments become affordable and available to the millions of patients with cancer, particularly those living in resource-constrained environments.
